# The Impact of Sugar-Sweetened Beverage Consumption on the Liver: A Proteomics-Based Analysis

**DOI:** 10.3390/antiox9070569

**Published:** 2020-07-01

**Authors:** Janina Benade, Lucien Sher, Sheneez De Klerk, Gaurang Deshpande, Dirk Bester, Jeanine L. Marnewick, Gary Sieck, Ismail Laher, M. Faadiel Essop

**Affiliations:** 1 Center for Cardio-Metabolic Research in Africa (CARMA), Department of Physiological Sciences, Stellenbosch University, Stellenbosch 7600, South Africa; janinabenade@gmail.com (J.B.); 18718078@sun.ac.za (L.S.); sheodk@gmail.com (S.D.K.); gaurang1712@gmail.com (G.D.); ismail.laher@ubc.ca (I.L.); 2 Applied Microbial and Health Biotechnology Institute, Cape Peninsula University of Technology, Bellville 7535, Cape Town, South Africa; besterd@cput.ac.za (D.B.); MarnewickJ@cput.ac.za (J.L.M.); 3 Faculty of Health and Wellness Sciences, Cape Peninsula University of Technology, Bellville 7535, Cape Town, South Africa; 4 Department of Physiology and Biomedical Engineering, Mayo Clinic, Rochester, MN 55905, USA; sieck.gary@mayo.edu; 5 Department of Anesthesiology, Pharmacology & Therapeutics, University of British Columbia, Vancouver, BC V6T 1Z3, Canada

**Keywords:** sugar-sweetened beverages, metabolic syndrome, endoplasmic reticulum stress, mitochondrial dysfunction, mitochondrial fission and fusion, antioxidant capacity, calcium homeostasis

## Abstract

Cardiometabolic complications such as the metabolic syndrome and Type 2 Diabetes Mellitus (T2DM) are major causes of global morbidity and mortality. As sugar-sweetened beverages (SSBs) are implicated in this process, this study aimed to obtain greater mechanistic insights. Male Wistar rats (~200 g) were gavaged with a local SSB every day for a period of six months while the control group was gavaged with an iso-volumetric amount of water. Experimental dosages were calculated according to the surface area-to-volume ratio and were equivalent to 125 mL/day (in human terms). A proteomic analysis was performed on isolated liver samples and thereafter, markers of endoplasmic reticulum (ER) stress, antioxidant/oxidant capacity, calcium regulation, and mitochondrial functionality were assessed. These data show that SSB consumption resulted in (a) the induction of mild hepatic ER stress; (b) altered hepatic mitochondrial dynamics; and (c) perturbed calcium handling across mitochondria-associated ER membranes. Despite significant changes in markers of ER stress, the antioxidant response and calcium handling (proteomics data), the liver is able to initiate adaptive responses to counteract such stressors. However, the mitochondrial data showed increased fission and decreased fusion that may put the organism at risk for developing insulin resistance and T2DM in the longer term.

## 1. Introduction

Cardiometabolic complications such as metabolic syndrome and Type 2 Diabetes Mellitus (T2DM) pose major global burdens of morbidity and result in ~19 million deaths annually [1]. Moreover, their prevalence rapidly increased over the past few decades and they continue to rise, especially in low- and middle-income countries. Here, excess sugar-sweetened beverage (SSB) intake is emerging as a crucial mediator of such pathology [2] as it is linked with hyperglycemia, dyslipidemia, hyperinsulinemia, and weight gain [3,4]. In support, increased SSB intake is associated with suboptimal lifestyle choices (reduced physical activity, poor dietary choices) [5] and the development of insulin resistance and T2DM [6,7]. As SSBs contain excessive amounts of rapidly absorbable carbohydrates such as glucose and high-fructose corn syrup [8], the glycemic load on the liver sharply increases following its consumption. Thus, regular SSB consumption and an increased metabolic load can lead to hepatocyte dysfunction that can present in the form of endoplasmic reticulum (ER) stress, which, in turn, is strongly implicated in T2DM onset [9,10,11].

Endoplasmic reticulum stress occurs due to a disequilibrium between ER load and protein-folding capacity and is characterized by the accumulation of unfolded or misfolded proteins in the ER lumen [12,13,14,15]. Well-known causes of ER stress include increased metabolic load, mitochondrial dysfunction, disturbed calcium handling, and elevated reactive oxygen species (ROS) levels [16,17]. The ER-associated degradation (ERAD) pathway and the unfolded protein response (UPR) are two important pathways that can be upregulated to salvage hepatocyte functionality and restore intracellular homeostasis [18]. Here, the ERAD pathway facilitates the translocation of terminally misfolded proteins into the cytoplasm to be degraded by the ubiquitin-proteasome pathway (UPP) [19]. When the ERAD pathway is unable to alleviate the accumulation of mutant proteins, the UPR simultaneously promotes the translocation of unfolded proteins and enhances ER protein-folding capacity by increasing the number of protein-folding chaperones [13]. However, its prolonged upregulation and/or dysfunction may result in the UPR becoming pro-apoptotic in nature [9].

Perturbed mitochondrial functionality and morphology are both inducers and downstream consequences of ER stress [15,20]. The ER and the mitochondria are closely associated at junctions known as mitochondria-associated ER membranes (MAMs) [21,22]. These organelles are primary sources of intracellular calcium and poor MAM structural integrity results in mitochondrial dysfunction and improper interorganellular calcium handling [13,23]. Perturbations in calcium homeostasis are also implicated in ER stress [24]. Mitochondria-associated ER membranes contain a variety of regulatory and channel proteins such as mitofusin 2 (MFN2), glucose-regulated protein 75 (grp75), inositol-1,4,5-triphosphate receptor (IP3R) and voltage-dependent anion-selective channel (VDAC) [23]. While MFN2 is important for mitochondrial fusion, it also tethers the mitochondrion to the ER and thus directly contributes to MAM structure and function [25,26]. Previous research found that poor mitochondrial functionality and decreased hepatic MFN2 expression are associated with increased hepatic gluconeogenesis and reduced insulin sensitivity [24]. These findings highlight the important role that MAMs play in modulating ER and mitochondrial functionality, and by mediating calcium transfer between these organelles.

Despite the large amount of epidemiological data supporting the link between SSB consumption and the onset of T2DM, the underlying mechanisms driving this process remain relatively unexplored [27]. In light of this, we established a unique rat model of long-term SSB consumption [28]. Here, we employ relatively low SSB dosages in order to generate data that will be more applicable to the broader population and also to detect the earliest perturbations underlying disease onset. For the current study, male rats were gavaged with a local SSB (every day) for a period of six months and compared to matched controls. A proteomic analysis was subsequently performed on isolated liver tissues and thereafter selected markers of ER stress, calcium, antioxidant capacity and mitochondrial functionality were assessed. 

## 2. Methods and Materials 

### 2.1. Animals and Experimental Protocol

Male Wistar rats (weighing ~200 g at start of experiment) were daily gavaged with a well-known, local SSB (Jive^©^) for a 6-month period. At the time, this product contained 13 g sugar (sucrose) per 100 mL. The rats were exposed to a 12-h day/night cycle and had *ad libitum* access to standard chow and water. They were housed three per cage and familiarized with the environment, handlers, and techniques for a week prior to the start of experimental procedures.

The experimental dosage of Jive^©^ was calculated according to their respective group and current weight. The SSB group (*n* = 8) received a dosage that was equivalent to 125 mL (~54 calories) of beverage for an adult person that weighs ~60 kg. Here the surface area-to-volume ratio of the rat was used to calculate the volume required for various weight categories [29]. The control group (*n* = 8) was gavaged with an iso-volumetric amount of water. Jive^®^ was selected as the SSB of choice since it is locally produced and relatively inexpensive compared to other brands, making it a popular option for the South African population. The high sugar content also allowed for smaller volumes to be gavaged to ensure animal welfare. This study was conducted with the permission of the Animal Ethics Committee of Stellenbosch University (South Africa) (Ethics # SU-ACUM13-00012) and all procedures and animal handling were in agreement with the Guide for the Care and Use of Laboratory Animals of the National Academy of Science (NIH publication No. 85-23, revised 1996).

### 2.2. Tissue Collection 

As this was a sub-study (of a larger one) these data have been previously published by us, i.e., the percentage weight gain and also serum levels for: glucose (fasted), glycosylated hemoglobin (HbA1c) and uric acid [28]. By the end of the 6-month period, rats were sedated with 5% isoflurane and pedal reflexes tested before they were euthanized. Liver tissues were carefully dissected out, snap-frozen in liquid nitrogen and stored at −80°C for subsequent analyses. 

### 2.3. Proteomics

In brief, 100 μg tissue was homogenized in 0.5 mL extraction buffer. The lysates were subsequently centrifuged at 12,000× *g* and ice-cold acetone added to the supernatant in a 1:4 ratio. Proteins were precipitated overnight at −20°C, where after pellets were separated, cleaned and protein concentrations spectrophotometrically determined. Proteins were thereafter digested in a trypsin solution, dried and resuspended. Residual digest reagents were subsequently removed, and the samples prepared for liquid chromatography and mass spectrometry analysis. Data were acquired and analyzed using ScaffoldQ+ software (Proteome Software, Portland, OR, USA). Peptides and proteins were validated with the X!Tandem search algorithm and the Peptide and Protein Prophet algorithms. 

### 2.4. Isolation of the Endoplasmic Reticulum

An ER isolation kit (Sigma-Aldrich, St. Louis MO, USA) was employed to extract and isolate the ER from homogenized hepatic tissue. Isotonic Extraction Buffer^®^ was added to slices of liver tissue and homogenized. The homogenate was then differentially centrifuged at 1000× *g* for 10 min and then at 12 000× *g* for 15 min. Following the addition of calcium chloride to the supernatant, the sample was centrifuged again at 8000× *g* for 10 min to obtain rough ER-enriched microsomes. The pellet was then re-suspended in Isotonic Extraction Buffer^®^. The entire isolation procedure was carried out at 4°C.

### 2.5. Calcium Colorimetric Assay

A calcium assay (Sigma-Aldrich, St. Louis MO, USA) was used to determine hepatic calcium concentrations by measuring the absorbance of the chromogenic complex that forms between calcium ions and *o*-cresolphthalein. This analysis was completed for the whole cell homogenate and the ER fraction, respectively. Thereafter Chromogenic Reagent^®^ together with a 5 mM Calcium Standard Solution^®^ were pipetted into each well of a 96-well plate. Calcium Assay Buffer^®^ was then added and the reaction incubated for 5–10 min at room temperature. During this time, the plate was protected from light exposure. Absorbance was then measured at 570 nm using a microplate reader. 

### 2.6. Western Blotting

Western blotting techniques were used to assess markers of mitochondrial function, i.e., MAM integrity, the degree of ER stress as well as intracellular calcium levels. Dynamin-related protein 1 (DRP1) (fission marker), MFN2 (fusion marker), and peroxisome proliferator-activated receptor gamma coactivator 1-alpha (PGC1α) were selected as markers of mitochondrial function. The expression of IP3R and VDAC1 was measured to gain insight into MAM composition. The three primary sensors of ER stress, namely inositol requiring enzyme 1 (IRE1), PKR-like-eukaryotic initiation factor 2 alpha kinase (PERK) and activating transcription factor 6 (ATF6), as well as C/EBP homologous protein (CHOP), a downstream marker of ER stress, were chosen to elucidate the extent of UPR upregulation. Lastly, we investigated the expression of calpain and cytochrome c as markers for dysregulation of cytosolic and intramitochondrial calcium levels, respectively. 

Proteins for each experimental group were extracted using a radio-immunoprecipitation assay buffer (Cell Signaling Technology, Danvers, MA, USA) and then separated on denaturing PAGE gels (50 µg per well, Criterion Gel System; Bio-Rad, Hercules CA; either 10% or 4–15% gradient gels). Protein expression was detected with anti-DRP1 (DNM1L, ab56788), anti-MFN2 (ab50843), anti-PGC1α (ab106814), anti-IP3R (Cell Signaling Technology^®^, Danvers, MA, USA, 8568), anti-porin (VDAC1, ab15895), anti-IRE1 (ab48187) anti-PERK (ab79483), anti-ATF6 (Novus Biologicals, Centennial, CO, USA, 70B1413.1), anti-CHOP (Cell Signaling Technology^®^, 2895), anti-calpain-1 large subunit (Cell Signaling Technology^®^, 2556) and anti-cytochrome c (Cell Signaling Technology^®^, 6H2.B4). Primary antibody was detected using horseradish peroxidase-conjugated secondary antibody, and signals were developed using Supersignal West Dura Chemiluminescent Substrate (Pierce Chemical Co., Rockford, IL, USA). Band intensities for MFN2, DRP1, PGC1α, and VDAC1 were normalized against expression of glyceraldehyde 3-phosphate dehydrogenase (GAPDH; MilliporeSigma, Burlington, MA, USA) and the rest to total protein. Note, all images of Western blotting data are shown in the Supplemental Figures S1–S3.

### 2.7. Oxidative Stress Analyses 

All of the assays below were done in triplicate.

#### 2.7.1. Thiobarbituric Acid Reactive Substances (TBARS)

Liver samples were homogenized in Buffer A (50 mM NaPO4, 1 mM EDTA, pH 7.5) as in previous experiments. The supernatant (50 μL) was vortexed with 6.25 μL butylated hydroxytoluene (BHT) (4 mM in ethanol), and 50 μL ortho-phosporic acid (0.2 M). Thiobarbituric acid reagent (6.25 μL; 0.11M in 0.1M NaOH) was added and the samples were vortexed for 10 s. The samples were placed in a heating bath for 45 min at 90 °C followed by 2 min on ice, and 5 min at room temperature. N-Butanol (500 μL) and saturated NaCl (50 μL) were added to each sample before another 10 s of vortexing. Samples were centrifuged at 13,700× *g* for 2 min and 300 μL of the top phase were added to the wells. The plate was thereafter read at 532 nm (25 °C) using a SpectraMax i3x platform plate reader (Molecular Devices, China). Systemic TBARS were also measured by using 50 μL of plasma instead of 50 μL of liver homogenate supernatant. 

#### 2.7.2. Glutathione Redox Status 

This protocol was adapted from Asensi et al. (1999) [30]. Briefly, liver samples (~200 μg) were homogenized on ice in 2 mL Buffer A (50 mM NaPO4, 1 mM EDTA, ph 7.5). Reduced glutathione (GSH) and oxidized (GSSG) samples were prepared separately. For GSSG analysis an additional 10 μL 1-methyl-2-vinyl-pyridinium trifluoromethane sulfonate (30 mM in 0.1 M hydrochloric acid) was added to every 1 mL Buffer A. Samples were centrifuged at 15,000× *g* for 5 min and the supernatants were diluted (10–40×) and used for analysis. A standard curve was prepared using GSH (3 μM) and GSSG (1.5 μM) stock solutions and Buffer A. All other reagents were also made up in Buffer A. 50 μL of each standard and sample (each in triplicate) were added to the microtiter plate. Thereafter we added 50 μL 0.3 mM 5,5′Dithiobis-(2-nitrobenzoic acid) to each well followed by 50 μL glutathione reductases (0.02 U/μL). The plate was incubated for 5 min at room temperature after which 50 μL NADPH (1 mM) was added to each well. The absorbance was measured directly to obtain a reading every 30 s for a total period of 5 min on a SpectraMax i3x platform plate reader (Molecular Devices, China) set at 25 °C and 412 nm. 

#### 2.7.3. Conjugated Dienes (CDs)

Liver samples (~100 μg) were homogenized in 2 mL methanol, and 1 mL chloroform was added to the lysates. Lysates were vortexed briefly before it was centrifuged at 3000× *g* for 1 min to allow for separation. Of note, for samples that did not separate 100 μL saturated NaCl was added and such samples re-centrifuged. The bottom layer of the samples was pipetted into new microtubes and left open overnight at 4°C to dry out. On the second day 700 μL cyclohexane was added to each of the microtubes after it was vortexed. Subsequently, 200 μL of sample was added to a 96-well plate and the absorbance was read on a SpectraMax i3x platform plate reader (Molecular Devices, China) set at 25 °C and 232 nm using cyclohexane as a blank. All samples were assayed in triplicate. 

#### 2.7.4. Oxygen Radical Absorbance Capacity (ORAC)

Liver samples were homogenized in Buffer A as described above. Thereafter it was centrifuged at 12,000× *g* for 10 min. The supernatant was collected and deproteinized by centrifuging 50 μL of 0.25 M perchloric acid and 50 μL supernatant together at 14,000× *g* for 15 min. The resultant supernatant was diluted (2–4×) and 12 μL added to the black 96-well plate. Next, 138 μL of fluorescein (working solution) was added to each well followed by the addition of 50 μL (25 mg/mL) of 2,22-Azobis (2-methylpropionamidine) dihydrchloride to each well after which the plate was read on a fluorometer (Floroskan Asent, Thermoscientifc, SA) at 485 nm, with readings taken every minute for a 2-h period. 

### 2.8. Statistical Analysis

All statistical analyses were conducted using Graphpad Prism v7.04 (Graphpad Software Inc., San Diego CA) and all data are presented as mean ± standard error of the mean (SEM). The Shapiro-Wilk test was used to test for normality. Differences between groups were further analyzed by means of a two-tailed T-test. Outliers were tested for using the Grubbs’ statistical test. Protein quantitation was performed by first performing an ANOVA analysis and applying the Hochberg-Benjamini correction (significant when *p* < 0.00275 after correction). The proteins shown to be statically different were marked for fold change calculations. For the biochemical antioxidant analyses a value of *p* < 0.05 indicated significance. 

## 3. Results

We previously published phenotypic data for this model and some of the major changes induced by 6 months of SSB consumption included: increased body weight gain, together with higher systemic uric acid and HbA1c levels [28]. However, we found no significant differences between the SSB group and controls for the homeostasis model assessment of insulin resistance.

### 3.1. Proteomics Analysis of the Liver

The proteomic analysis was performed on both the Control and SSB groups (*n* = 6) with each sample analyzed in duplicate. A total of 3379 proteins were detected in the samples at a false discovery rate of 0.5% and 222 proteins were uniquely regulated in one of the groups versus the other. Of these, the 122 proteins that exhibited a probability reading >95% were considered for further analysis.

Collectively, we found a marked increase in the expression of proteins involved in protein folding (P13084, Q66HD0, TIM44, D3ZUU5, D3ZX38), protein degradation (VCIP1, A1AT, UFD1, Q66HD0, O88321, D3ZFY8) and oxidative phosphorylation (Q5BJZ3, NDUV2, SDHB, ATP5H). (Table 1A). Together these changes are indicative of early adaptive responses to ER stress. We also detected the altered expression of proteins involved in regulating calcium homeostasis (B5DEQ0, D3ZP47, RGN and TCTP), antioxidant capacity (THTM, D3ZEN5, GSTA1, GSTA3, GSTA6, Q498E0, THIOM) and mitochondrial function (NADC, THTM) (Table 1B ). These changes could be involved in the onset of ER stress. Lastly, we noted a decreased expression of proteins involved in pyruvate and glucose metabolism (Q5BJX2 and Q5RKL4) and lipid metabolism (LYPA1, Q711G3), but elevated levels of proteins involved in lipid and cholesterol storage (Q641Z6, Q65ZS7, HMCS1, P11915) (Table 1C). The remainder of the proteins that exhibited altered expression due to SSB consumption are described in Supplementary Tables S1 and S2.

### 3.2. Markers of ER Stress

**Figure 1:**
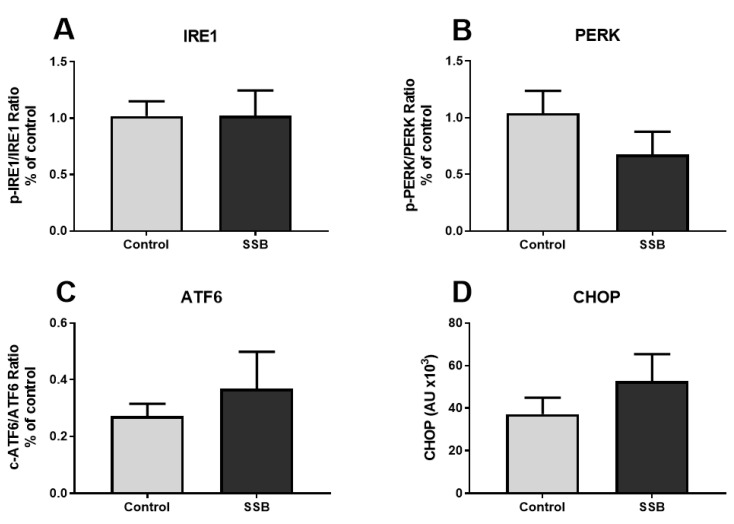
The effect of sugar-sweetened beverage (SSB) consumption on known markers of endoplasmic reticulum (ER) stress. (**A**) Inositol requiring enzyme 1 (IRE1), (**B**) PKR-like-eukaryotic initiation factor 2 alpha kinase (PERK), (**C**) activating transcription factor 6 (ATF6), and (**D**) C/EBP homologous protein (CHOP). Data are displayed as mean ± SEM (*n* = 8).

To further investigate the degree of ER stress demonstrated by the proteomic data, Western blotting techniques were employed to measure the expression of ER stress-sensors IRE1, PERK, and ATF6. We also analyzed the expression of CHOP, a pro-apoptotic downstream marker. Data are reported as a ratio of activated (phosphorylated/cleaved) protein over total protein for the ER stress-sensors IRE1, PERK, and ATF6. Although the proteomic data pointed towards a phenotype of ERAD and UPR upregulation, we found no significant differences in expression of the typical markers of ER stress (Figure 1). 

### 3.3. Markers of Mitochondrial Function and MAM Composition

**Figure 2:**
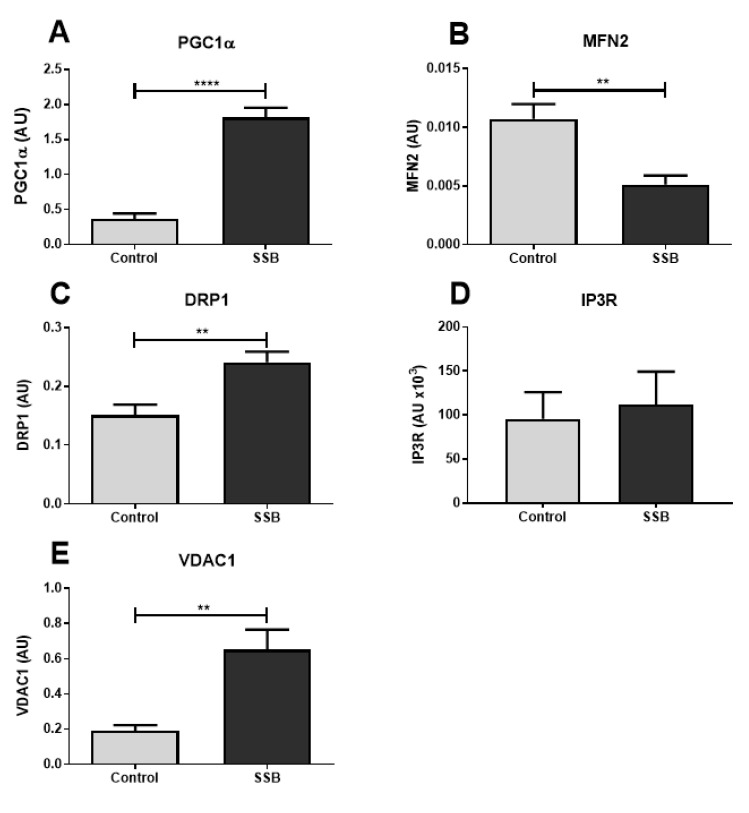
The impact of SSB consumption on mitochondrial markers. (**A**) Peroxisome proliferator-activated receptor gamma coactivator 1-alpha (PGC1), (**B**) mitofusin 2 (MFN2), (**C**) dynamin-related protein 1 (DRP1), (**D**) inositol-1,4,5-triphosphate receptor (IP3R), and (**E**) voltage-dependent anion-selective channel 1 (VDAC1). Data are displayed as mean ± SEM. ** *p* < 0.01; **** *p* < 0.001 (*n* = 8).

As improper mitochondrial function is associated with ER stress [31,32], we determined the expression of markers of mitochondrial fission and fusion, as well as mitochondrial biogenesis. We also measured the expression of proteins found within ER-mitochondrial junctions as they are known to influence mitochondrial function and calcium handling. A robust elevation of hepatic PGC1α levels were detected in the SSB group versus controls (*p* < 0.0001) (Figure 2A). We also observed a concurrent decrease in MFN2 (*p* = 0.0037) and increase in DRP1 (*p* = 0.0049) expression in the SSB group versus the control (Figure 2B,C, respectively). Although there were no changes in IP3R expression, the SSB group displayed higher VDAC1 levels compared to the control (*p* = 0.0031) (Figure 2E). 

### 3.4. Calcium Assessments

**Figure 3:**
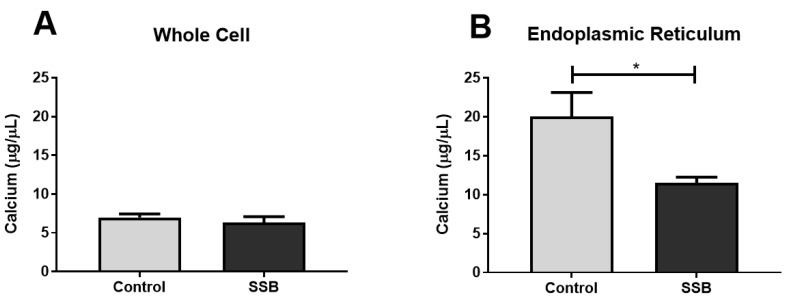
Hepatic calcium levels in response to SSB intake. (**A**) whole cell homogenate, and (**B**) isolated ER fraction. Data are displayed as mean ± SEM. * *p* < 0.05 (*n* = 8).

**Figure 4:**
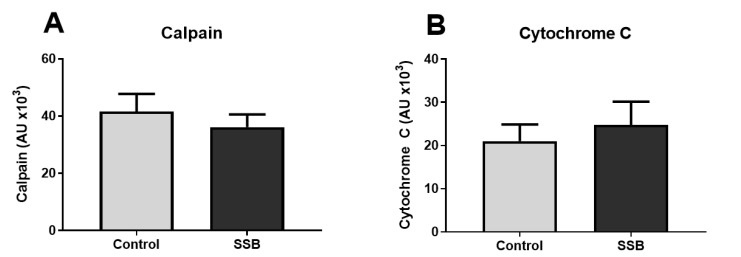
The effect of SSB consumption on calpain and cytochrome c expression. (**A**) Calpain and (**B**) cytochrome c levels. Data are displayed as mean ± SEM (*n* = 8).

Calcium is an essential intracellular messenger and ER stress is often coupled with a dysregulation in calcium homeostasis [33]. Our data reveal that calcium levels were not significantly different between the SSB and the control groups (Figure 3A). However, for the ER fraction, the SSB group exhibited lower calcium levels versus controls (Figure 3B) (*p* = 0.013). There were no significant differences between the SSB and controls for calpain and cytochrome c levels (Figure 4).

### 3.5. Oxidative Stress Analyses

**Figure 5:**
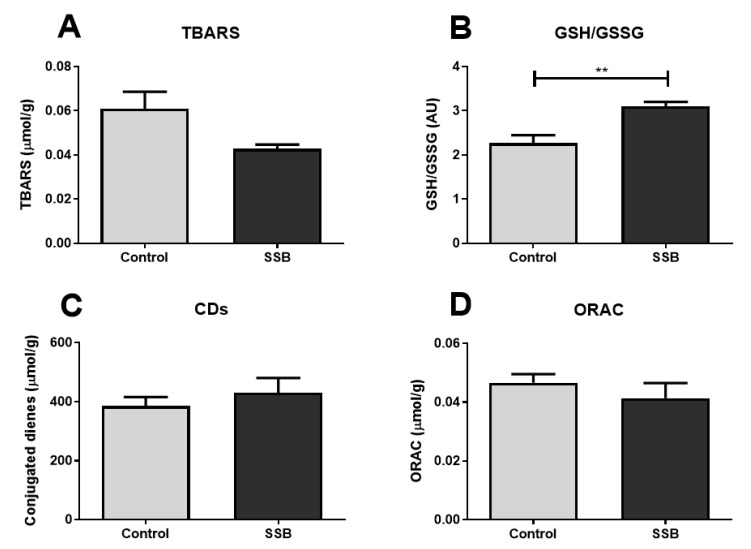
The impact of SSB consumption on hepatic oxidative stress markers. (**A**) Thiobarbituric acid reactive substances (TBARS), (**B**) reduced glutathione (GSH)/oxidized glutathione (GSH/GSSG) ratio, (**C**) conjugated dienes (CDs), and (**D**) oxygen radical absorbance capacity (ORAC). Data are displayed as mean ± SEM. ** *p* < 0.01 (*n* = 8).

A combination of assays that measured antioxidant defense systems and markers for ROS-induced damage were employed to gain insight into the overall hepatic oxidative status. There were no significant differences between the SSB group and controls for the oxidative lipid damage markers, TBARS, and CDs nor for the antioxidant capacity marker, ORAC (Figure 5). However, the SSB group exhibited an increased GSH/GSSG ratio versus the control group (*p* = 0.004) (Figure 5B). 

## 4. Discussion

As the underlying mechanisms driving SSB-mediated cardiometabolic complications remain poorly understood, we employed a unique in-house developed *in-vivo* rat model of SSB intake to assess the effects of 6-months consumption on the liver. The main findings from moderate but frequent SSB intake are as follows: (a) the induction of mild hepatic ER stress; (b) altered hepatic mitochondrial dynamics and (c) perturbed calcium handling across MAMs. 

### 4.1. SSB Consumption Induced a Proteomic Phenotype Indicative of Mild Hepatic ER Stress

To our knowledge, this is the first time a proteomic analysis has been completed to assess SSB-mediated effects on the liver. The data showed that several classes of proteins were altered following SSB intake, e.g., mitochondrial structural integrity and function, antioxidant/oxidant regulators, and ER-related changes. Hepatic ER stress is characterized by the accumulation of unfolded or misfolded proteins. Our data shows upregulation of the ERAD and UPR pathways as indicated by increased expression of several proteins involved in protein degradation and folding (Table 1A). These pathways are triggered during the early adaptive phase following the onset of ER stress. The proteomic data also shows an upregulation of mitochondrial proteins involved in oxidative phosphorylation. This likely also represents an adaptive mechanism to ER stress as others found augmentation of mitochondrial function and oxidative phosphorylation in order to increase ATP production as part of such a response [14]. In support, enhanced mitochondrial metabolism following transient ER stress was also observed in mammalian adrenal glands and gonads [34]. 

ER stress can be triggered by a variety of cellular stressors, such as an increased metabolic burden, changes in mitochondrial metabolism, elevated ROS levels, and deficient calcium handling [20,35]. In addition to the markers of ER stress, the proteomic data also elucidated possible mechanisms involved in the onset of ER stress in the context of SSB consumption (Table 1B). Our findings revealed a decreased expression of proteins with antioxidant capacity as well as proteins that alter the availability of NADPH which subsequently influences glutathione levels (Table 1B). This is in contrast to others investigating hepatic protein expression of hamsters consuming a high fructose diet where they found an upregulation in the expression of peroxiredoxin- and glutathione S-transferase isoforms, suggested as part of a protective response [36]. This discrepancy in these two specific proteins may be due to the unique properties of fructose compared to sucrose, or because fructose was consumed in a solid form versus liquid in our model. Further oxidative stress analysis revealed an increase in the GSH/GSSG ratio in the SSB group which suggests that a similar protective mechanism may have been triggered in our model. The proteomic data also suggests that SSB consumption may alter calcium handling and mitochondrial function. 

Finally, the proteomics data also revealed changes in the expression of proteins involved in glucose, fructose and lipid metabolism (Table 1C). We detected a marked downregulation of phosphoglycerate mutase 1 (an enzyme of the glycolytic pathway) and Pdhx protein (structural protein of the pyruvate dehydrogenase complex) levels, suggesting the attenuation of glucose metabolism. This is in accordance with evidence showing that ER stress can suppress hepatic gluconeogenesis [14]. However, the most drastic changes were observed in proteins involved in lipid metabolism. Collectively such data shows a decrease in proteins involved in fatty acid catabolism together with increased cholesterol synthesis and storage. This is in accordance with others reporting on the influence of SSBs on fructose-mediated lipogenesis in the liver [37,38]. As the metabolic changes associated with ER stress have not yet been fully elucidated, it remains unclear whether such alterations observed in metabolic proteins are induced by the SSB *per se* or whether it is a downstream consequence of ER stress, or a combination of both. 

In the context of T2DM and insulin resistance, other studies have found similar protein expression profiles. For example, Morand et al. (2005) studied the expression of ER-related proteins in insulin-resistant hamsters on a high fructose diet and found the expression of chaperone proteins and other proteins involved in protein folding were comparably altered [39]. Others found the onset of diabetes in Goto-Kakizaki rats triggered an increase in the expression of mitochondrial proteins, particularly those involved in oxidative phosphorylation, while antioxidants were suppressed [40]. The similarity between our data and that of insulin-resistant/diabetic models likely indicates that moderate (but frequent) SSB consumption induced early diabetic characteristics in our model despite the fact that it is not yet reflected in the fasting blood glucose levels (data not shown, refer [28]). Furthermore, these studies support the idea that ER stress and mitochondrial metabolism may be implicated in the onset of early hepatic pathophysiology.

We next sought to confirm the presence of ER stress by quantifying the relative expression of the three ER stress sensors, namely: IRE1, PERK and ATF6. We also measured CHOP expression as it has been identified as a reliable marker for the detection of sustained UPR upregulation [41,42]. However, we observed no significant differences between the SSB and control group for the ER stress markers. By contrast, others reported marked differences in the expression of a variety of ER stress markers (IRE1, PERK, and CHOP). Notably, this study also observed similar changes elicited by a high-fructose and high-fat diet [43]. Others suggested that downstream targets of ER stress may represent a more robust approach for the detection of UPR activity [19]. Here, the authors indicated that the detection of phospho-IRE1, phospho-PERK and the proteolytic activation of ATF6 by the Golgi apparatus may be difficult due to their relatively low levels of expression [19]. This may help explain the lack of observed differences we found for several ER stress markers. Studies have also discerned that changes in mitochondrial function and metabolism induced a phenotype of ER stress [20,32]. This suggests that the phenotype of SSB-induced ER stress may be a direct result of altered mitochondrial dynamics. 

### 4.2. SSB Consumption Elicited Changes in Mitochondrial Dynamics

The data showed that frequent SSB consumption promoted mitochondrial biogenesis together with increased and decreased mitochondrial fission and fusion, respectively. It is now well established that perturbations in mitochondrial function, mass, and metabolism are implicated in the progression to insulin resistance, a hallmark feature of T2DM [44,45]. PGC1α is one of the master regulators of mitochondrial biogenesis and influences hepatic glucose metabolism through the co-activation of transcription factors that control important enzymes of the gluconeogenic pathway. In the context of obesity and T2DM, others demonstrated upregulated PGC1α expression that in turn promoted hepatic glucose production [46,47]. Elevated PGC1α levels are also associated with ER stress and upregulated mitochondrial fission [31]. 

The balance between mitochondrial fission and fusion is largely influenced by nutrient availability, with downregulated fusion symptomatic of nutrient abundance [48]. Our findings demonstrate a shift away from mitochondrial fusion and towards fission which is suggestive of a pathophysiologic outcome. In support, mitochondrial fission can participate in both hyperglycemia-induced mitochondrial fragmentation and ROS-mediated apoptosis [49]. Moreover, others demonstrated decreased MFN2 levels, mitochondrial fragmentation, and increased ROS production in rodents fed a high-fat diet (HFD) or exposed to excess glucose availability [48,50,51,52]. Reduced hepatic MFN2 levels also resulted in ER stress, which in turn upregulated hepatic glucose production and impaired insulin signaling [31]. By contrast, MFN2 overexpression ameliorated HFD-induced insulin resistance by targeting insulin signaling pathways [53]. The role of inflammation-induced ER stress with downstream effects on MFN2 and mitochondrial function is also of increasing interest [54]. For example, the pro-inflammatory modulator tumor necrosis factor-alpha selectively activated the IRE1α/X-box binding protein-1 pathway in a dose- and time-dependent fashion [55].

Mitofusin 2 is essential for MAM integrity as it is responsible for tethering the ER to mitochondria and for facilitating interorganellular calcium transfer. For example, studies showed that MFN2 deficiency leads to impaired mitochondrial calcium uptake due to an increased distance between the ER and mitochondria [26]. Together with grp78, IP3R, and VDAC1 it allows for the efflux of calcium through the ER membrane, across the interorganellular space, and via the outer mitochondrial membrane [23]. Notably, IP3Rs are the primary calcium-release channels of the ER membrane and are regulated by IP3 and calcium itself [56]. Although we found no changes in IP3R expression, there was a marked increase in hepatic VDAC1 expression in the SSB group versus controls. VDAC1 is highly permeable to calcium, mediates calcium entry into the inter-membrane space and regulates permeability transition pore activity [57]. Increased VDAC1 expression therefore suggests that mitochondrial calcium levels were likely to be elevated. 

### 4.3. Moderate SSB Consumption Perturbed Calcium Handling Across MAMs

Calcium homeostasis is crucial for cell growth and metabolism [14]. In the context of diabetes and obesity, adequate mitochondrial calcium uptake is fundamental for insulin-mediated glucose uptake [44]. We found that six months of regular SSB consumption significantly lowered calcium levels (in ER fraction) versus controls. This is in accordance with others who showed that ER-related calcium release resulted in the activation of DRP1-dependent mitochondrial fission [33]. Increased mitochondrial fission can also downregulate cellular respiratory rates and glucose uptake. Others also found that the efflux of ER-related calcium can increase both cytosolic and mitochondrial calcium concentrations [58]. 

To gain further insight into intramitochondrial and cytosolic calcium levels, we measured the expression of cytochrome c and calpain. Here, the rationale was that elevated intramitochondrial calcium concentrations can induce cytochrome c release and increased cytosolic calcium levels are known to act on calpain; both able to trigger apoptosis via caspase activation [33,59]. Cytochrome c can also bind with IP3Rs to further promote efflux of calcium into mitochondria to thereby enhance apoptotic signaling [60]. However, we found no significant differences between the SSB and control groups for calpain and cytochrome c expression. This suggests that the SSB-induced calcium ER efflux was perhaps not severe enough to elicit a pro-apoptotic shift. These findings are also in agreement with the proteomic data which was not indicative of any significant apoptotic signaling. 

**Figure 6:**
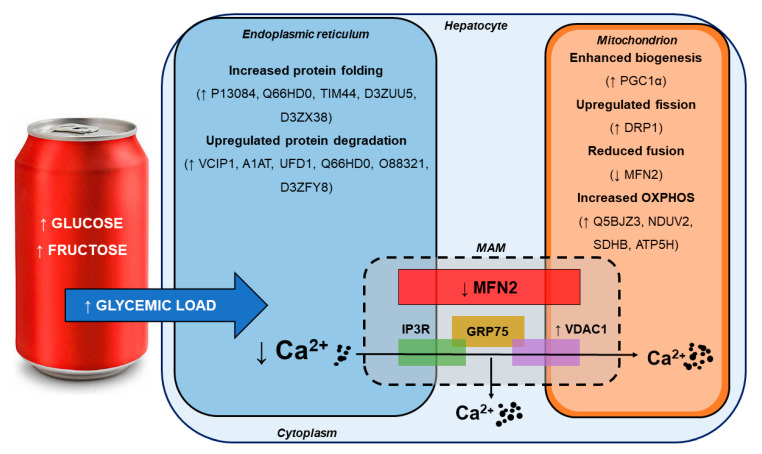
Summary of findings demonstrating SSB-mediated effects on hepatic ER stress and its impact on the mitochondrion.

In summary, our data reveal that 6-months of SSB consumption resulted in significant changes in several protein classes as demonstrated by the proteomics analysis. Here, markers of ER stress, the antioxidant response, calcium handling, and mitochondrial-related function were markedly altered and thus suggest early strain on hepatic metabolism and function. While the liver seems able to initiate adaptive responses to largely counteract such stress at this relatively early stage of SSB intake, we observed perturbed calcium handling across MAMs that led to a significant decrease in ER calcium levels. ER-related calcium release is associated with increased mitochondrial fission (and decreased fusion) that may put the organism at risk for developing the metabolic syndrome and T2DM in the longer term (Figure 6). 

## Figures and Tables

**Table 1:** SSB-induced changes in protein expression (listed according to function).

Name (Accession Number)	Function; Location	SSB vs. Control
(A) Signs of ER stress (elevated protein folding, protein degradation and oxidative phosphorylation)		
**Cluster of DnaJ (Hsp40) homolog subfamily B member 1 (predicted) isoform CRA_a (D3ZUU5)**	Chaperone cofactor-dependent protein folding; cytosol, extracellular vesicular exosome and nucleus (ScaffoldQ+).	Inf (absent in Control)
**Prefoldin 1 (D3ZX38)**	Binds specifically to cytosolic chaperonin to promote protein folding; ER (Prefoldin complex) (ScaffoldQ+).	280% ↑
**Cluster of mitochondrial import inner membrane translocase subunit TIM44 (TIM44)**	Involved in importing proteins from the mitochondrial inner membrane to the mitochondrial matrix (ATP-dependent), chaperone binding; mitochondrial inner membrane and matrix (ScaffoldQ+; UniProtKB).	160% ↑
**Cluster of nucleophosmin (sp|P13084|NPM)**	Involved in various cellular processes including protein chaperoning; cytoplasm and nucleus (UniProtKB).	80% ↑
**Endoplasmin (sp|Q66HD0|ENPL)**	Involved in protein chaperoning and ER-associated degradation; ER lumen (UniProtKB).	40% ↑
**Ubiquitin fusion degradation protein 1 homolog (UFD1)**	Partakes in the degradation ER-associated degradation and ubiquitin fusion degradation of misfolded proteins and the activation of certain transcription factors; cytosol and nucleus (UniProtKB).	140% ↑
**Cluster of Protein LOC100912618 (tr|D3ZFY8|D3ZFY8)**	Catalyzes the attachment of ubiquitin protein to proteins. Also involved in various steps of DNA replication; cytoplasm and nucleus (ScaffoldQ+).	50% ↑
**Deubiquitinating protein VCIP135 (VCIP1)**	Deubiquitination of proteins to prevent protein degradation; ER and golgi stacks (UniProtKB).	95% ↓
**Alpha-1-antiproteinase (A1AT)**	Associated with the acute-phase response and inhibits proteases activity; secreted into intracellular space (ScaffoldQ+; UniProtKB).	30% ↓
**Nicotinamide nucleotide transhydrogenase (Q5BJZ3)**	NADPH regeneration and proton transport; mitochondrial inner membrane (ScaffoldQ+).	390% ↑
**Cluster of NADH dehydrogenase [ubiquinone] flavoprotein 2 mitochondrial (sp|P19234|NDUV2)**	Mitochondrial electron transport, NADH to ubiquinone; mitochondrial ETC complex I (ScaffoldQ+).	30% ↑
**Succinate dehydrogenase [ubiquinone] iron-sulfur subunit (SDHB)**	Subunit of Complex II → transfer electrons from succinate to ubiquinone; mitochondrial inner membrane (UniProtKB).	20% ↑
**ATP synthase subunit d (sp|P31399|ATP5H)**	Maintenance of ATP synthase structure during ATP synthesis; mitochondrial inner membrane (UniProtKB).	20% ↑
(B) Possible mechanisms of ER stress (altered calcium handling, redox balance and mitochondrial function)		
**Cluster of translationally-controlled tumor protein (TCTP)**	Involved in cellular calcium homeostasis and inhibits apoptotic process signaling; cytoplasm and extracellular exosome (UniProtKB).	40% ↑
**Translocon-associated protein subunit β (B5DEQ0)**	Involved in ER calcium homeostasis (STRING); ER membrane (ScaffoldQ+).	100% ↓
**Phosphohistidine phosphatase 1 isoform CRA_a (D3ZP47)**	Dephosphorylates proteins and inhibits calcium channels; cytosol and extracellular exosome (ScaffoldQ+).	80% ↓
**Regucalcin (RGN)**	Cellular calcium ion homeostasis, cytoplasm and nucleoplasm (ScaffoldQ+).	20% ↓
**Cluster of thioredoxin domain-containing protein 12 (sp|Q498E0|TXD12)**	Involved in redox homeostasis and inhibits ER stress-induced apoptosis; lumen of ER (UniProtKB).	460% ↑
**Thioredoxin. mitochondrial (THIOM)**	Involved in cellular redox homeostasis and mitochondrial membrane potential. Mitochondrial thioredoxin is also involved in inhibiting apoptosis; mitochondria (UniProtKB).	50% ↑
**Cluster of protein RGD1565183 (D3ZJD3)**	Translation - structural component of large ribosomal subunit (ScaffoldQ+); nucleolus (UniProtKB).	80% ↓
**Cluster of glutathione S-transferase-α1 (GSTA1)**	Protects against oxidative stress and products of lipid peroxidation through glutathione peroxidase activity, cytosol and extracellular exome (NCBI; UniProtKB).	50% ↓
**Cluster of peroxiredoxin-5 [mitochondrial] (D3ZEN5)**	Antioxidant properties; mitochondria (STRING).	40% ↓
**Nicotinate-nucleotide pyrophosphorylase [carboxylating] (NADC)**	Involved in quinolinate catabolism and nicotinamide adenine dinucleotide (NAD^+^) synthesis. NAD+ plays a role in cellular metabolism and mitochondrial health (ScaffoldQ+).	60% ↓
**3-mercaptopyruvate sulfurtransferase (THTM)**	Involved in the production of the antioxidant hydrogen sulfide (H_2_S). Changes in enzyme activity and H_2_S is indicative of the development of T2DM and hyperglycemia-induced epithelial cell damage; cytoplasm and mitochondria (UniProtKB).	40% ↓
(C) Possible downstream consequences of ER stress - changes in pyruvate glucose and lipid metabolism		
**Cluster of Pdhx protein (Q5BJX2)**	Important structural protein of pyruvate dehydrogenase complex; mitochondria (ScaffoldQ+).	70% ↓
**Cluster of dimethylglycine dehydrogenase (tr|Q5RKL4)**	Catabolizes dimethylglycine to glycine; mitochondrial matrix Dimethylglycine is involved in the regulation of glucose metabolism (UniProtKB)	70% ↓
**Acyl-protein thioesterase 1 (LYPA1)**	Involved in fatty acid metabolism and protein depalmitoylation; cytoplasm (ScaffoldQ+, UniProtKB).	80% ↓
**Cluster of isoamyl acetate-hydrolyzing esterase 1 homolog (sp|Q711G3|IAH1)**	Lipid catabolism (lipase) and hydrolase activity; extracellular exosome (UniProtKB).	40% ↓
**Cluster of EH domain-containing protein 1 (sp|Q641Z6|EHD1)**	Regulates cholesterol homeostasis and lipid droplet storage. Also involved in endocytosis; cytoplasm and endocytic vesicles (ScaffoldQ+).	1000% ↑
**Rat apolipoprotein E protein (Q65ZS7)**	Facilitates the binding and uptake of lipoprotein particles (LDLs in particular) to clear it out of the plasma (UniProtKB).	100% ↑
**Hydroxymethylglutaryl-CoA synthase. cytoplasmic (HMCS1)**	Involved in the initial phase of cholesterol biosynthesis; cytoplasm, nucleoplasm and plasma membrane (UniProtKB).	40% ↑
**Cluster of non-specific lipid-transfer protein (sp|P11915|NLTP)**	Regulates intracellular cholesterol transport; peroxisome (ScaffoldQ+).	10% ↑

## Supplementary Materials

The following are available online at https://www.mdpi.com/2076-3921/9/7/569/s1, Figure S1: The effect of SSB consumption on known markers of ER stress – Western blotting; Figure S2: The impact of SSB consumption on mitochondrial markers – Western blotting; Figure S3: The effect of SSB consumption on calpain and cytochrome c expression – Western blotting; Table S1: Proteins exhibiting an SSB-induced decrease in expression (listed from highest to lowest); Table S2: Proteins exhibiting an SSB-induced increase in expression (listed from highest to lowest).
