# Supplementary material for: The Impact of Sugar-Sweetened Beverage Consumption on the Liver: A Proteomics-Based Analysis
Source: Antioxidants (Basel). 2020 Jul 1;9(7):569. doi: 10.3390/antiox9070569 (PMC7402188; doi:10.3390/antiox9070569)
Supplement: Supplementary file 1 [file antioxidants-09-00569-s001.zip › Supplementary Data Tables S1 and S2 15 June 2020_Clean.docx]

**Table S1:** **Proteins exhibiting an SSB-induced decrease in expression (listed from highest to lowest).**

| **Name (accession number)** | **Function; location** | **Percentage decrease (SSB vs Control)** |
| --- | --- | --- |
| **3-hydroxyisobutyryl-CoA hydrolase [mitochondrial] (HIBCH)** | Partakes in valine, leucine and isoleucine degradation catabolism; mitochondria (ScaffoldQ+). | 100% ↓ |
| **Protein Cmc1 (D3ZTN2)** | Involved in the assembly and function of cytochrome C oxidase (COX); mitochondria (Complex IV) (ScaffoldQ+). | 100% ↓ |
| **Protein Tjp3 (D3Z8G7)** | Structural component of tight junctions; between cells (UniProtKB) | 100% ↓ |
| **Bile acid-CoA:amino acid N-acyltransferase (BAAT)** | Important enzyme in step in the bile acid biosynthetic process before bile is released into canaliculi; cytosol and peroxisomes (ScaffoldQ+).  Could regulate intracellular levels of fatty acids through its effects on acyl-CoA (UniProtKB). | 100% ↓ |
| **Cluster of LUC7-like (G3V9R0)** | Involved in RNA splicing; nucleus (ScaffoldQ+). | 100% ↓ |
| **Heat shock protein β8 (HSPB8)** | Chaperone activity (response to temperature stress); Golgi apparatus, cytoplasm, nucleoplasm (UniProtKB). | 100% ↓ |
| **ATPase inhibitor, mitochondrial (ATIF1)** | Protects cell against ATP-depletion when mitochondrial membrane potential drops below a critical threshold; mitochondrial proton-transporting ATP synthase complex (ScaffoldQ+; UniProtKB). | 99% ↓ |
| **Enthoprotin (Q6DGF2)** | Initiates the assembly of clathrin for clathrin-coated endocytosis vesicles; Golgi apparatus (UniProtKB). | 98% ↓ |
| **Lymphocyte specific 1 isoform CRA_a (Q4QQV6)** | Involved in signal transduction and chemotaxis; extracellular exosome and cell membrane (ScaffoldQ+). | 91% ↓ |
| **Cluster of isoform 2 of myelin basic protein (sp\|P02688-2\|MBP)** | Possibly involved in the formation of transcriptional complexes; nucleus (UniProtKB). | 90% ↓ |
| **Calponin-3 (CNN3)** | Involved in epithelial cell differentiation and smooth muscle contractions; cytoplasm and cytoskeleton (ScaffoldQ+, UniProtKB). | 80% ↓ |
| **Cluster of biliverdin reductase B Flavin reductase (NADPH) (B5DF65)** | Reduces biliverdin to bilirubin with the concomitant oxidation of a nicotinamide adenine dinucleotide (NADH) or nicotinamide adenine dinucleotide phosphate (NADPH) cofactor; cytoplasm (UniProtKB) | 80% ↓ |
| **Cluster of nipsnap1 protein (Q5EBA4)** | Involved in pain sensation, cell surface and mitochondrial inner membrane (ScaffoldQ+). | 80% ↓ |
| **Cluster of clathrin light chain B (sp\|P08082\|CLCB)** | Intracellular protein transport; membrane of clathrin-coated endocytic vesicles and trans-Golgi network (ScaffoldQ+). | 80% ↓ |
| **Cluster of tetratricopeptide repeat protein 36 (TTC36)** | Possibly involved in protein folding, also known as HSP70-binding protein 21 (UniProtKB). Subcellular location unknown. | 70% ↓ |
| **Cluster of 1,2-dihydroxy-3-keto-5-methylthiopentene dioxygenase (MTND)** | Step 5 of methionine salvage pathway (UniProtKB); cytosol (Reactome). | 70% ↓ |
| **Cluster of annexin A3 (ANXA3)** | Promotes angiogenesis and involved in immune responses; cytoplams, cell membrane, extracellular exomes (ScaffoldQ+; UniProtKB). | 70% ↓ |
| **Ddx17 protein (Q568Z8)** | Involved in the regulation of transcription; nucleus. | 70% ↓ |
| **Cluster of GM2 ganglioside activator (Q6IN37)** | Regulates the degradation of GM2 gangliosides (UniProtKB); mitochondrion (ScaffoldQ+). | 70% ↓ |
| **Cluster of LOC689593 (B2RYA8)** | ER-associated protein catabolism; cytosol and nucleus (ScaffoldQ+; UniProtKB). | 60% ↓ |
| **Cluster of 60S ribosomal protein L7 (B0K031)** | Translation - structural constituent large ribosome; cytosol (ScaffoldQ+). | 60% ↓ |
| **Cluster of sorbin and SH3 domain-containing protein 2 (tr\|F1LPM3\|F1LPM3)** | Involved in the formation of stress fibers and cell adhesion; perinuclear region of cytoplasm (ScaffoldQ+).  . | 60% ↓ |
| **Cluster of uncharacterized protein (F1LRJ9)** | Intragolgi protein transport (ScaffoldQ+, STRING); nucleus, cytosol, golgi membrane (UniProtKB). | 60% ↓ |
| **Cluster of tubulin-specific chaperone A (TBCA)** | Post-chaperonin tubulin folding pathway (ScaffoldQ+); cytoskeleton (UniProtKB). | 60% ↓ |
| **Cluster of aflatoxin B1 aldehyde reductase member 2 (sp\|Q8CG45\|ARK72)** | Aldehyde reductase activity (requires NADPH) and toxin degradation; Golgi apparatus and cytoplasm (UniProtKB). | 50% ↓ |
| **Cluster of NADH dehydrogenase [ubiquinone] 1-α subcomplex subunit 6 (tr\|D4A3V2\|D4A3V2)** | Involved in electron transport from NADH to the ETC; mitochondrial subunit of complex I of ETC (UniProtKB). | 50% ↓ |
| **Cluster of 60S ribosomal protein L26 (tr\|G3V6I9\|G3V6I9)** | Translation - structural component of large cytosolic ribosomes (ScaffoldQ+). | 50% ↓ |
| **Cluster of histone H3 (D3ZJ08)** | Inhibits transcription from RNA polymerase II promoter and involved in nucleosome assembly; nucleus (ScaffoldQ+; UniProtKB). | 50% ↓ |
| **Cluster of clathrin light chain A (sp\|P08081\|CLCA)** | Intracellular protein transport (clathrin-mediated endocytosis); membrane of clathrin-coated endocytic vesicles and trans-Golgi network (ScaffoldQ+). | 40% ↓ |
| **Cluster of 40S ribosomal protein S8 (tr\|M0R7Q5\|M0R7Q5)** | Translation - small ribosomal subunit; cytosol and cell membrane (ScaffoldQ+; UniProtKB). | 40% ↓ |
| **Cluster of protein Taf15 (D3ZSS1)** | Involved in binding of nucleotides and the regulation of transcription, nucleoplasm (ScaffoldQ+). | 40% ↓ |
| **Cluster of N-acetylneuraminic acid synthase (B1WC26)** | Carbohydrate biosynthetic process, cytosol (ScaffoldQ+). | 40% ↓ |
| **Cluster of Protein LOC680161 (D4A6B9)** | Ribosome biogenesis and RNA binding, cytosolic large ribosomal subunit (ScaffoldQ+; UniProtKB). | 40% ↓ |
| **Cluster of sulfotransferase family cytosolic 1B member 1 (sp\|P52847\|ST1B1)** | Involved in the differentiation of epithelial cells; cytoplasm (UniProtKB). | 30% ↓ |
| **Cluster of uncharacterized protein (D4ADL2)** | Involved in translation - peptidyl-serine phosphorylation, nucleus (ScaffoldQ+). | 30% ↓ |
| **Cluster of ubiquinone biosynthesis protein COQ9 [mitochondrial] (COQ9)** | Partakes in the biosynthesis of coenzyme Q/ubiquinone; mitochondrial inner membrane (UniProtKB). | 30% ↓ |
| **Cluster of ATP synthase subunit delta. mitochondrial (G3V7Y3)** | Proton-transporting ATPase activity, rotational mechanism, mitochondrial inner membrane (UniProtKB). | 30% ↓ |
| **Cluster of phosphoglycerate mutase 1 (sp\|P25113\|PGAM1)** | Catalyzes step 8 of glycolytic pathway, cytosol (ScaffoldQ+). | 30% ↓ |
| **Cluster of D-β-hydroxybutyrate dehydrogenase [mitochondrial] (sp\|P29147\|BDH)** | Synthesis of ketone bodies; mitochondrial matrix (ScaffoldQ+). | 30% ↓ |
| **Cluster of 40S ribosomal protein SA (RSSA)** | Involved in small ribosomal subunit assembly and maintenance; plasma membrane (ScaffoldQ+). | 30% ↓ |
| **Cluster of GTPase activating protein (SH3 domain) binding protein 2 (tr\|Q6AY21\|Q6AY21)** | Nucleotide binding, cytoplasm (UniProtKB). | 20% ↓ |
| **Cluster of protein LOC100912599 (tr\|D3ZCZ9\|D3ZCZ9)** | Transports electrons from NADH to ubiquinone; mitochondrial ETC complex I (ScaffoldQ+). | 20% ↓ |
| **Cluster of 3-α-hydroxysteroid dehydrogenase (sp\|P23457\|DIDH)** | Synthesis of bile acids and bile salts; cytoplasm (Reactome; ScaffoldQ+). | 20% ↓ |
| **Coiled-coil-helix-coiled-coil-helix domain containing 3 isoform CRA_a (D3ZUX5)** | Involved in maintenance of inner mitochondrial membrane and suppress transcription from RNA polymerase II promoter, mitochondria and nucleus (UniProtKB). | 20% ↓ |
| **D-dopachrome decarboxylase (DOPD)** | Cytoplasm. | 20% ↓ |
| **Omega-amidase (NIT2)** | Converts potentially toxic metabolic intermediates to beneficial metabolic substrates; cytoplasm (UniProtKB). | 20% ↓ |
| **Cluster of 40S ribosomal protein S18 (RS18)** | Translation and ribosome biogenesis; cytoplasm (ScaffoldQ+, UniProtKB). | 20% ↓ |
| **Cluster of histone H1.4 (H14)** | Inhibits transcription from RNA polymerase II promoter and involved in nucleosome assembly, nucleus (ScaffoldQ+; UniProtKB). | 10% ↓ |
| **Cluster of Fumarylacetoacetase (FAAA)** | Amino acid catabolism; cytosol (UniProtKB). | 10% ↓ |

**Table S2:** **Proteins exhibiting an SSB-induced increase in expression (listed from highest to lowest).**

| **Name (accession number)** | **Function; location** | **Percentage increase (SSB vs Control)** |
| --- | --- | --- |
| **Cluster of heterogeneous nuclear ribonucleoprotein H2 (HNRH2)** | Involved in pre-mRNAs processing; nucleoplasm (Reactome; STRING). | Inf  (Absent in Control group) |
| **RGD1307526 protein (Q568Z5)** | Function and location unknown. | Inf  (Absent in Control group) |
| **Crk-like protein (CRKL)** | Mediates intracellular signal transduction; cytosol, endosome and extracellular vesicular exosome (ScaffoldQ+; UniProtKB). | 530% ↑ |
| **Death-associated protein 1 (DAP1)** | Negatively regulates autophagy; nucleus and cytoplasm (UniProtKB). | 470% ↑ |
| **UPF0449 protein C19orf25 homolog (CS025)** | Function and location unknown. | 350% ↑ |
| **Cluster of protein Snrpd2 (B5DES0)** | Regulation mRNA splicing and snRNP assembly (Reactome); nucleus (UniProtKB). | 180% ↑ |
| **Cluster of Aa1018 (Q7TQ11)** | Structural protein that is involved in receptor-mediated endocytosis and immune response; extracellular vesicular exosome (ScaffoldQ+; UniProtKB). | 150% ↑ |
| **Cluster of heterogeneous nuclear ribonucleoprotein (HNRPF)** | Regulation of RNA splicing and mRNA processing; nucleoplasm and cytoplasm (ScaffoldQ+). | 120% ↑ |
| **Acidic leucine-rich nuclear phosphoprotein 32 family member B (AN32B)** | Cell-cycle regulation; nucleus (Reactome). | 120% ↑ |
| **Fetuin-B (FETUB)** | Secreted by hepatocytes and elevated in diabetic mice; extracellular region. Associated with impaired glucose and steatosis (Uniport). | 100% ↑ |
| **Cluster of bifunctional ATP-dependent dihydroxyacetone kinase/FAD-AMP lyase (cyclizing) (DHAK)** | Involved in fructose metabolism; cytosol (Reactome). Alternative name: Triose kinase (UniProtKB). | 80% ↑ |
| **Protein Snrnp70 (B2RZ74)** | Involved in mRNA splicing and gene expression; nucleoplasm and cytoplasm. (Reactome; UniProtKB). | 80% ↑ |
| **Cluster of protein Ybx2 (D4A3P0)** | Binding of nucleic acids; nucleus, nucleolus and cytoplasm (Uniport). | 70% ↑ |
| **Histidine-rich glycoprotein (HRG)** | Platelet activation and negative regulation of cell growth (ScaffoldQ+); secreted (UniProtKB). | 70% ↑ |
| **Cluster of multiple coagulation factor deficiency 2 (Q6GQY2)** | Vesicle mediated protein transport (Reactome; ScaffoldQ+); Golgi apparatus and ER (ScaffoldQ+). | 70% ↑ |
| **Cluster of plasminogen activator inhibitor 1 RNA-binding protein (sp\|Q6AXS5\|PAIRB)** | Partakes in the moduation of mRNA stability; cytoplasm and nucleus (UniProtKB). | 60% ↑ |
| **Cluster of heterogeneous nuclear ribonucleoprotein C (C1/C2) (tr\|G3V9R8\|G3V9R8)** | Involved in mRNA splicing and nucleotide binding; nucleus, nucleoplasm and extracellular exosome (ScaffoldQ+; UniProtKB). | 50% ↑ |
| **UBX domain-containing protein 1 (UBXN1)** | Negative regulator of protein degradation and nuclear factor kappa B (NF-κB) signaling; nucleus, cytoplasm and ER (UniProtKB). | 40% ↑ |
| **Thymosin β4 (TYB4)** | Regulation of cell migration and actin filament organization for tissue repair; cytoskeleton (ScaffoldQ+). | 40% ↑ |
| **Cluster of alcohol dehydrogenase 1 (ADH1)** | Reduces alcohol molecules to produce NADH; Cytoplasm (UniProtKB). | 40% ↑ |
| **Cluster of lamina-associated polypeptide 2, isoform β (LAP2)** | Regulation of transcription; ER membrane (ScaffoldQ+). | 40% ↑ |
| **Cluster of Hnrpa1 protein (Q6P6G9)** | Deoxyribonucleic acid (DNA) strand renaturation and mRNA transport; nucleoplasm and cytoplasm (ScaffoldQ+). | 40% ↑ |
| **Cluster of DnaJ homolog subfamily A member 1 (sp\|P63036\|DNJA1)** | Involved in protein transfer into mitochondria whereby it suppresses ER-associated apoptosis by inhibiting the translocation of Bax into mitochondria. Also inhibits c-Jun amino-terminal kinase (JNK) activity; ER and mitochondria (ScaffoldQ+; UniProtKB). | 30% ↑ |
| **Cluster of Phosphatidylethanolamine-binding protein 1 (PEBP1)** | Involved in the dephosphorylation of proteins and serine protease inhibition; cytoplasm, nucleus and extracellular exosome (ScaffoldQ+; UniProtKB). | 30% ↑ |
| **SP120 (Q63555)** | DNA and RNA binding; nucleus (STRING; UniProtKB). | 30% ↑ |
| **Protein mut (D3ZKG1)** | Propionyl-CoA catabolism to succinyl-CoA (intermediate of citric acid cycle); mitochondrial matrix (ScaffoldQ+). | 30% ↑ |
| **Beta-2-microglobulin (B2MG)** | Positive regulation of T cell-mediated cytotoxicity (stress signaling); Golgi apparatus (ScaffoldQ+). | 30% ↑ |
| **NADH dehydrogenase [ubiquinone] iron-sulfur protein 4 (sp\|Q5XIF3\|NDUS4)** | Subunit of Complex I - transfer electrons from NADH to the electron transport chain (ETC); mitochondrial inner membrane (UniProtKB). | 30% ↑ |
| **Transgelin (TAGL)** | Form cross-links with actin and possible marker for liver damage; cytoplasm (UniProtKB). | 20% ↑ |
| **Calcium-regulated heat stable protein 1 (CHSP1)** | Inhibit gluconeogenic gene expression; cytoplasm (UniProtKB). | 20% ↑ |
