# Supplementary figures and images for: The Impact of Sugar-Sweetened Beverage Consumption on the Liver: A Proteomics-Based Analysis
Source: Antioxidants (Basel). 2020 Jul 1;9(7):569. doi: 10.3390/antiox9070569 (PMC7402188; doi:10.3390/antiox9070569)

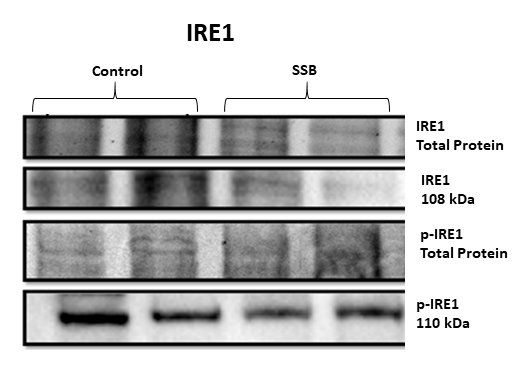

Supplement: Supplementary file 1 [file antioxidants-09-00569-s001.zip › Suppl Figure 1A IRE1 Western blot.TIF]

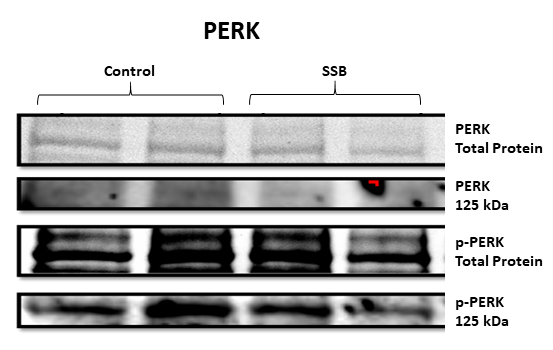

Supplement: Supplementary file 1 [file antioxidants-09-00569-s001.zip › Suppl Figure 1B PERK Western blot.tif]

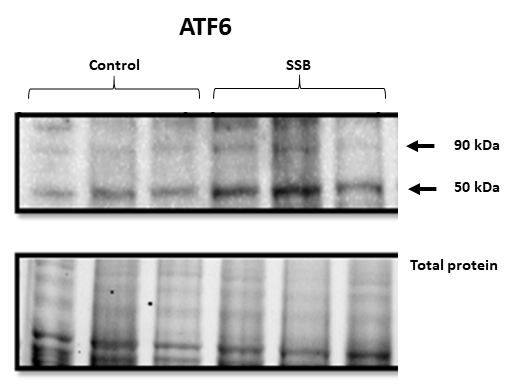

Supplement: Supplementary file 1 [file antioxidants-09-00569-s001.zip › Suppl Figure 1C ATF6 Western blot.TIF]

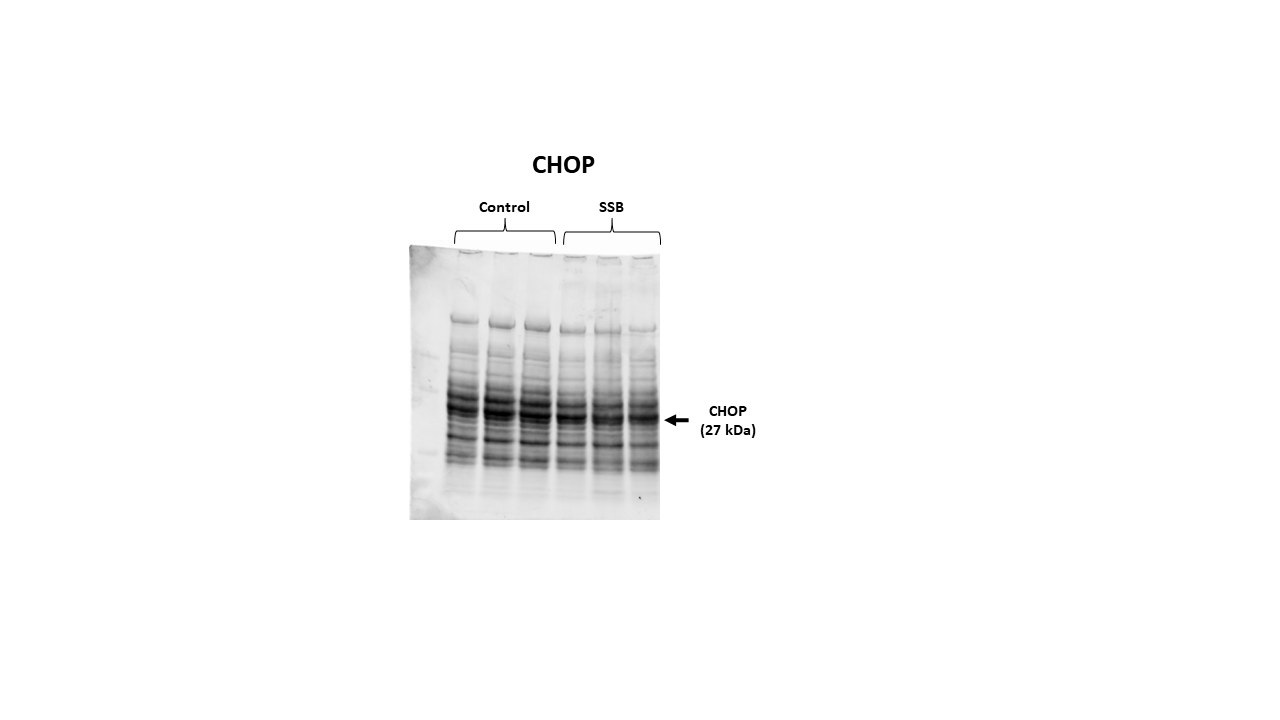

Supplement: Supplementary file 1 [file antioxidants-09-00569-s001.zip › Suppl Figure 1D CHOP Western blot.tif]

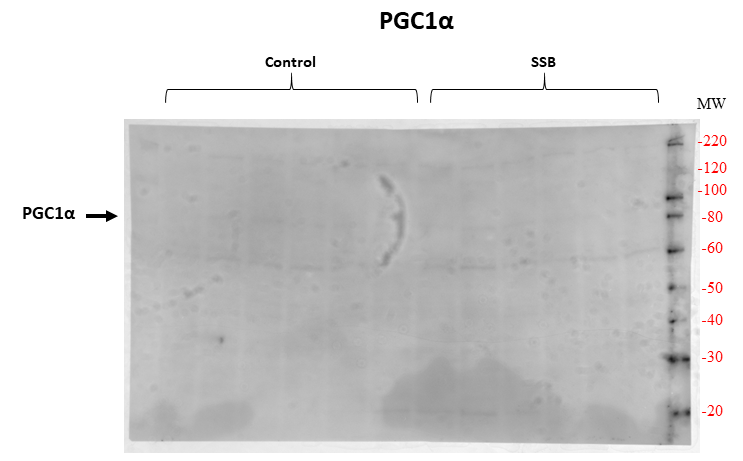

Supplement: Supplementary file 1 [file antioxidants-09-00569-s001.zip › Suppl Figure 2A PGC1 Western blot.TIF]

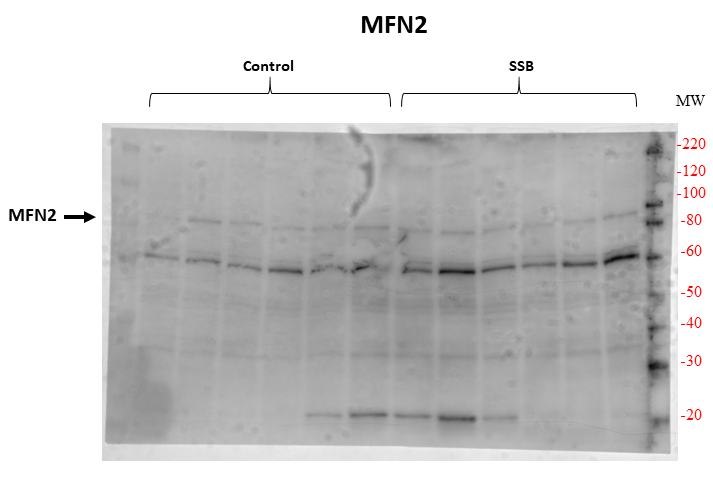

Supplement: Supplementary file 1 [file antioxidants-09-00569-s001.zip › Suppl Figure 2B MFN2 Western blot.TIF]

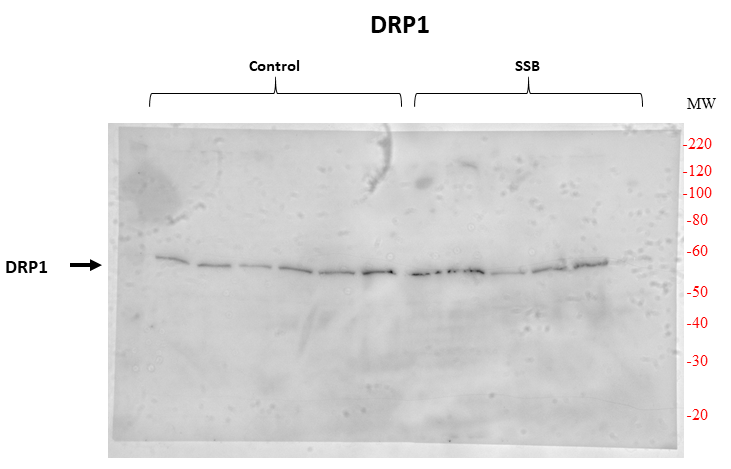

Supplement: Supplementary file 1 [file antioxidants-09-00569-s001.zip › Suppl Figure 2C DRP1 Western blot.TIF]

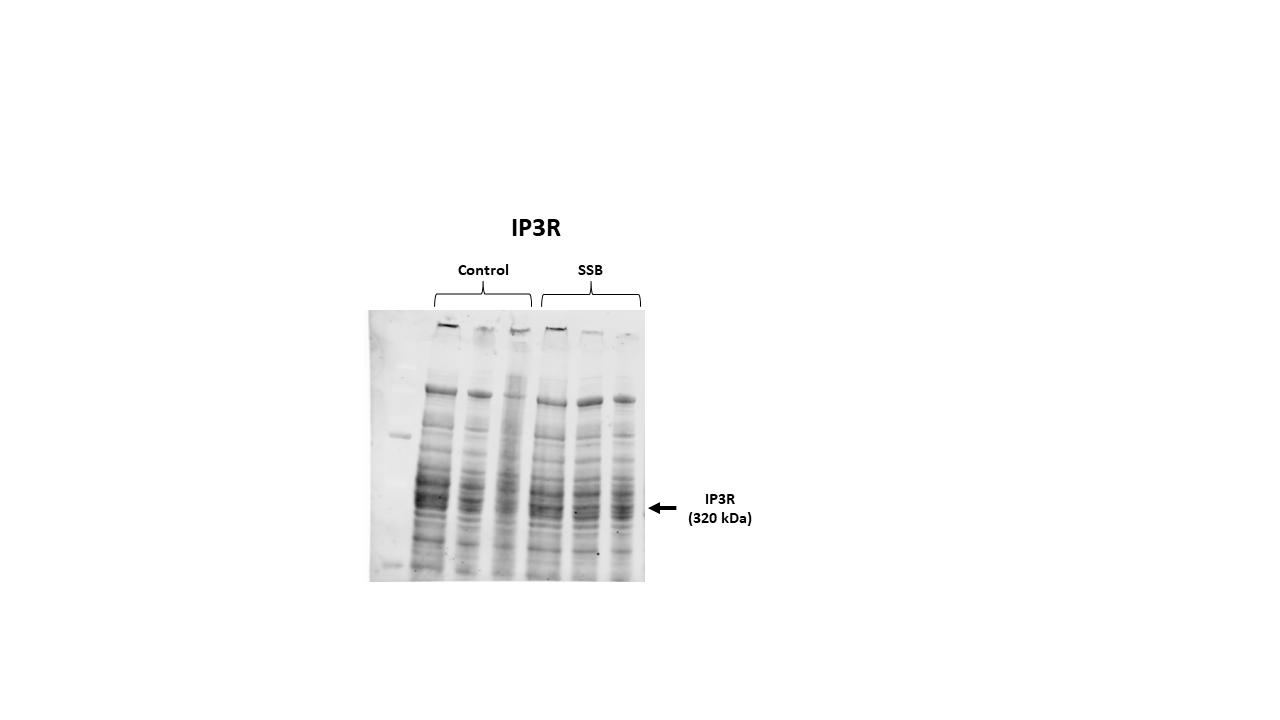

Supplement: Supplementary file 1 [file antioxidants-09-00569-s001.zip › Suppl Figure 2D IP3R Western blot.tif]

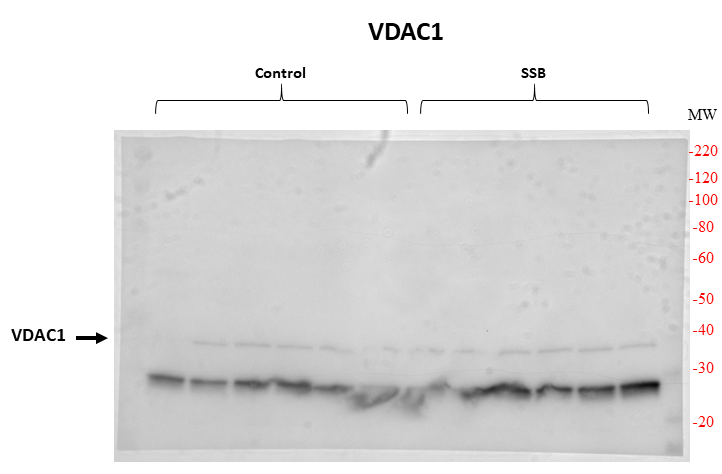

Supplement: Supplementary file 1 [file antioxidants-09-00569-s001.zip › Suppl Figure 2E VDAC1 Western blot.TIF]

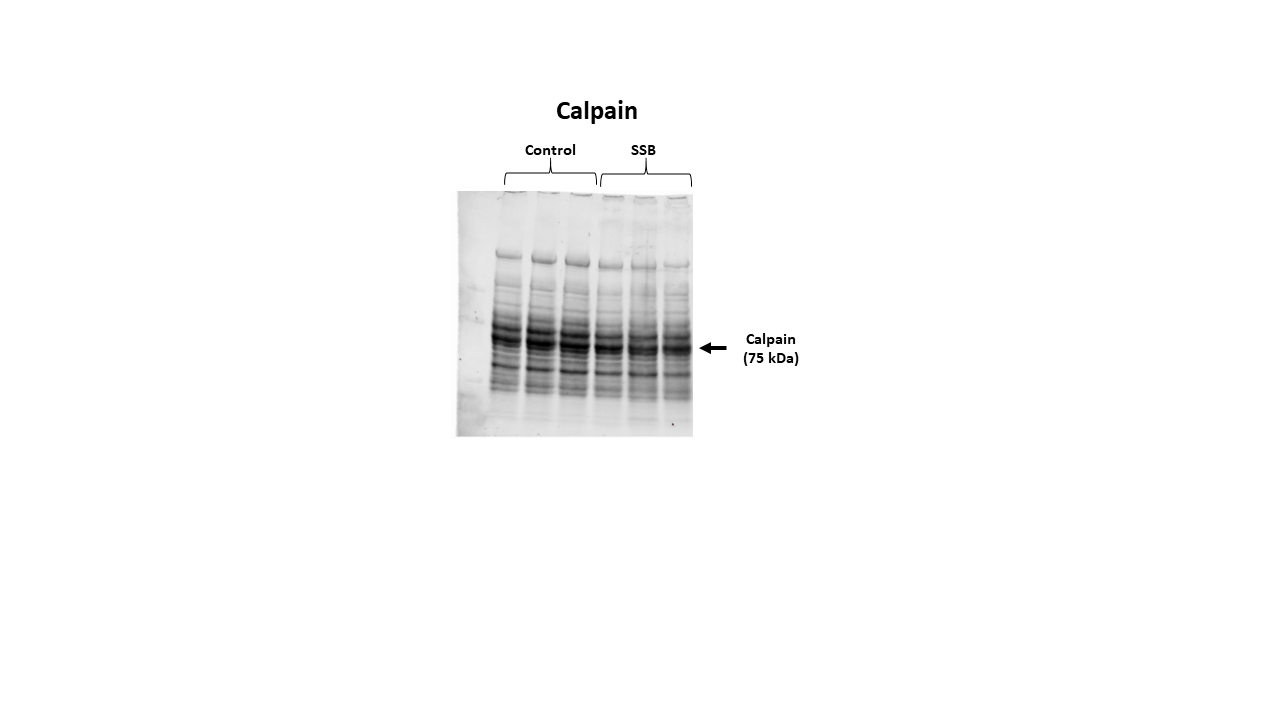

Supplement: Supplementary file 1 [file antioxidants-09-00569-s001.zip › Suppl Figure 3A Calpain Western blot.tif]

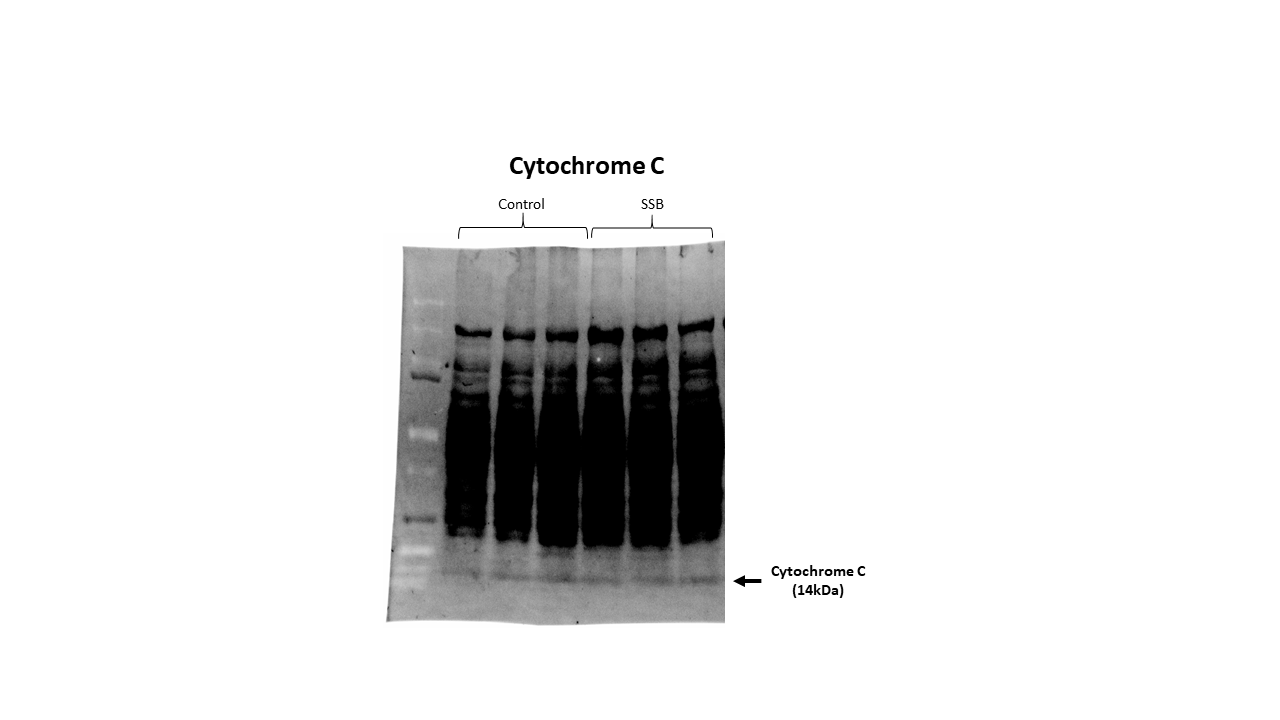

Supplement: Supplementary file 1 [file antioxidants-09-00569-s001.zip › Suppl Figure 3B Cytochrome C Western blot.TIF]
